# The Successful Synchronized Orchestration of an Investigator-Initiated Multicenter Trial Using a Clinical Trial Management System and Team Approach: Design and Utility Study

**DOI:** 10.2196/30368

**Published:** 2021-12-22

**Authors:** Dinesh Pal Mudaranthakam, Alexandra Brown, Elizabeth Kerling, Susan E Carlson, Christina J Valentine, Byron Gajewski

**Affiliations:** 1 University of Kansas Medical Center Kansas City, KS United States; 2 University of Cincinnati Cincinnati, OH United States

**Keywords:** data management, data quality, metrics, trial execution, clinical trials, cost, accrual, accrual inequality, rare diseases, healthcare, health care, health operations

## Abstract

**Background:**

As the cost of clinical trials continues to rise, novel approaches are required to ensure ethical allocation of resources. Multisite trials have been increasingly utilized in phase 1 trials for rare diseases and in phase 2 and 3 trials to meet accrual needs. The benefits of multisite trials include easier patient recruitment, expanded generalizability, and more robust statistical analyses. However, there are several problems more likely to arise in multisite trials, including accrual inequality, protocol nonadherence, data entry mistakes, and data integration difficulties.

**Objective:**

The Biostatistics & Data Science department at the University of Kansas Medical Center developed a clinical trial management system (comprehensive research information system [CRIS]) specifically designed to streamline multisite clinical trial management.

**Methods:**

A National Institute of Child Health and Human Development–funded phase 3 trial, the ADORE (assessment of docosahexaenoic acid [DHA] on reducing early preterm birth) trial fully utilized CRIS to provide automated accrual reports, centralize data capture, automate trial completion reports, and streamline data harmonization.

**Results:**

Using the ADORE trial as an example, we describe the utility of CRIS in database design, regulatory compliance, training standardization, study management, and automated reporting. Our goal is to continue to build a CRIS through use in subsequent multisite trials. Reports generated to suit the needs of future studies will be available as templates.

**Conclusions:**

The implementation of similar tools and systems could provide significant cost-saving and operational benefit to multisite trials.

**Trial Registration:**

ClinicalTrials.gov NCT02626299; https://tinyurl.com/j6erphcj

## Introduction

### Background

The evaluation of new treatment modalities through randomized controlled trials (RCTs) is the gold standard for advancing medical research; however, RCTs frequently fail to meet recruitment goals [1]. Multisite trials are frequently utilized to increase the availability of patients who meet inclusion criteria in phase 3 trials and increasingly in phase 1 and phase 2 trials as well. The benefits of performing multisite trials include more robust statistical analyses, reduced bias, expanded generalizability, and shorter recruitment periods [2,3]. However, multisite trials are far more difficult to perform effectively than single-site trials.

The primary research team and site leads have an obligation to ensure the trial is conducted in accordance with the International Council for Harmonization Guideline for Good Clinical Practice (ICH GCP) [4]. Standardized training and a central data management system are necessary to overcome the variability in clinical trial experience among study sites, which commonly have different recruiting practices, data entry processes, regulatory interpretation, and clinical trial experience [5,6]. Without standardization, these differences can threaten data harmonization, quality assurance, statistical analysis, data management, regulatory compliance, and recruitment [7]. A central data management system combined with standardized data entry protocols and customized reporting that includes ongoing accrual tracking [8,9] can facilitate data harmonization, quality assurance, data cleaning, and data analysis.

We detail here a clinical trial management system (CTMS), powered by WCG (Western Institutional Review Board–Copernicus Group, Inc) Velos and customized by the University of Kansas Medical Center (KUMC) Biostatistics & Data Science department to address the challenges of multisite trial management. The CTMS (comprehensive research information system [CRIS]) was customized to maximize team collaboration and produce effective workflow procedures for a phase 3, multisite RCT (the ADORE trial) in partnership with the Maternal and Child Health team at the Department of Dietetics and Nutrition at KUMC.

### The RCT in Brief

The ADORE (assessment of docosahexaenoic acid [DHA] on reducing early preterm birth) trial was a phase 3, investigator-initiated, adaptive-design, double-blind randomized, superiority trial designed to determine the potential of high dose DHA to reduce the incidence of early preterm birth (<34 weeks) (R01HD083292; HD NCT02626299). Pregnant women were assigned to either a standard prenatal DHA supplement of 200 mg per day or 1000 mg per day before 20 weeks gestation [10]. The study sought to enroll between 900 and 1200 pregnant women over a 4-year period, which lent itself naturally to a multisite configuration.

Three academic medical centers enrolled a total of 1100 participants in ADORE: the KUMC (n=489), University of Cincinnati (UC; n=252), and the Ohio State University (OSU; n=359). During the study, 128 employees were involved across the 3 trial teams.

While this paper focuses on a specific CTMS and RCT, the overall purpose is to characterize the strengths and weaknesses of a centralized data management system and its integration into multisite trials. The broad topics of database design, regulatory compliance, CTMS training and access, study management, and data management are emphasized throughout manuscript.

## Methods

### Methods Overview

The CTMS deployed at the KUMC was powered by WCG Velos and customized by the Biostatistics & Data Science department to include features related to randomization, automated reports, dashboards, etc, and titled the CRIS. Features such as electronic data capture, data monitoring, and data validation are common in clinical trial management systems. The value of the CRIS system was to standardize these aspects of clinical trial management systems for multiple sites, as well as introduce additional utility uniquely suited to multisite clinical trials.

### Development of an Electronic Case Report Form

Database design was an integral piece of the protocol development phase of study design. It was imperative to understand the capabilities of CRIS in collaboration with key personnel well before recruitment began. Critical factors to address included the number of study sites, sample size, number of treatment arms, and recruitment protocol. Senior members of the primary KUMC site team including principal investigators, the project director, biostatistician, and CTMS director of research information technology met regularly in person during the design phase. Primary, secondary, and tertiary data points were examined carefully, including their variable type and validation. Study personnel at satellite recruitment sites in Ohio additionally vetted data fields by verifying that the data elements that were part of the electronic case report form (eCRF) were aligned with the aims of the protocol. This ensured that these fields were able to capture the correct values for the study and allowed data capture to occur as categorical values rather than open text fields. Adverse event (AE)–reporting and participant-focused activity-tracking including standardized recruitment, randomization, blinding, treatment follow-up, and specimen collection forms were also considered in the CRIS database design prior to study start. An eCRF was an outcome of the design phase.

### Achieving Regulatory Compliance

In addition to the standard elements of ICH GCPs, eCRFs were required to be compliant with 21 CFR Part 11 *Electronic Records; Electronic Signature* to ensure security and accountability of study data [11,12]. Requirements of this compliance included controls for open systems, signature manifestations, record-linking, and controls for identification codes and passwords. Open system controls included continuous system validation, limitation of system access, computer-generated audit trails, operational system checks, accountability in record changes, document encryption, and several other controls. Signature controls ensured that any changes to records did not obscure previous information, that changes were associated the study member who made them, and that the date and time of the change was recorded. These controls, and several others, were integrated into CRIS development to ensure full CFR Part 11 compliance during electronic data capture.

### Study Personnel Training

The training of study personnel at all participating sites followed design of the eCRF. Both live and recorded sessions reviewed the protocol, the Manual of Operating Procedures, and the CRIS system and associated eCRF. Access to the system was provided only after users had completed both institutionally required ethics and study-specific training. Access within the system was role-based; that is, principal investigator, co-investigator, study coordinator, monitor, pharmacists, etc, to ensure study team members were only able to access data within CRIS relevant to their role on the trial. While the training itself was conducted outside of the CRIS through the use of prerecorded training videos and manual materials, study staff were tested on the material, and their testing results and training certification were documented within the CRIS system and was a prerequisite for system access.

The study operational director and primary principal investigator visited the participating sites before enrollment began, and the operational director made additional visits during the study to provide additional training and tips and to audit study documentation and workflow.

### Data Management

Data entry was performed at each site via the web-based browser and managed at KUMC through the Biostatistics & Data Science department. This was integrated to allow personnel at all sites to conserve time and labor typically allocated to data management. The benefit of eCRFs to data integrity, time management, and data analysis have been described [13,14].

The customized CRIS eCRFs were the primary repository for participant’s historical information obtained from either health records or participant self-reports. Individual forms used during the study included forms for patient characteristics, laboratory samples, primary outcome variables, and patient participation. Patient characteristics forms included assessments of health history, dietary supplement intake, maternal physical exam, and medical record review. These forms were completed during the enrollment visit after participants gave consent for the study.

Several eCRFs were utilized during the study including forms to record samples obtained (maternal blood draws, pregnancy labs, and urine collection) and to track results of pregnancy outcomes and estimated date of birth by study site. Lastly, forms detailing patient participation were integrated in the system including the signed informed consent, study coordinator sign off, forms for the delivery and tracking of treatment, withdrawal forms, and forms documenting adverse events. All forms could be viewed, selected, and copied in list form (Multimedia Appendix 1). Paper copies of source documents to verify data entry were retained by each study site and scanned copies submitted to CRIS for cross-validation and regulatory compliance.

### Randomization

The initial randomization table was generated by the statistical team in accordance with the study protocol guidelines. The data management team uploaded the randomization table under the CRIS. Every time a new participant was screened and deemed eligible, the participant at the time of enrollment was then randomized to a treatment arm on the basis of the stratification variables that were built under the randomization form. Once the randomization form was completed by the study coordinator the participant automatically would get randomized and the arm to which the participant was randomized would be displayed on the participants profile.

### Assuring Data Integrity

The accuracy of data entry was confirmed using a 2-pass approach whenever possible. Data entered into the eCRF and the source documentation uploaded as a secure file at satellite sites were accessible to team members at the main trial site who reviewed the eCRF for accuracy in comparison with the source document. In compliance with ICH GCP (version 4.9.1), study data were fully accessible to principal investigators responsible for evaluation of accuracy, completeness, and legibility of entered data. The ability to access the data was included to allow study coordinators to keep in contact with study participants at each site and quickly resolve any questions or concerns the study team or participant might have had.

**Figure 1:**
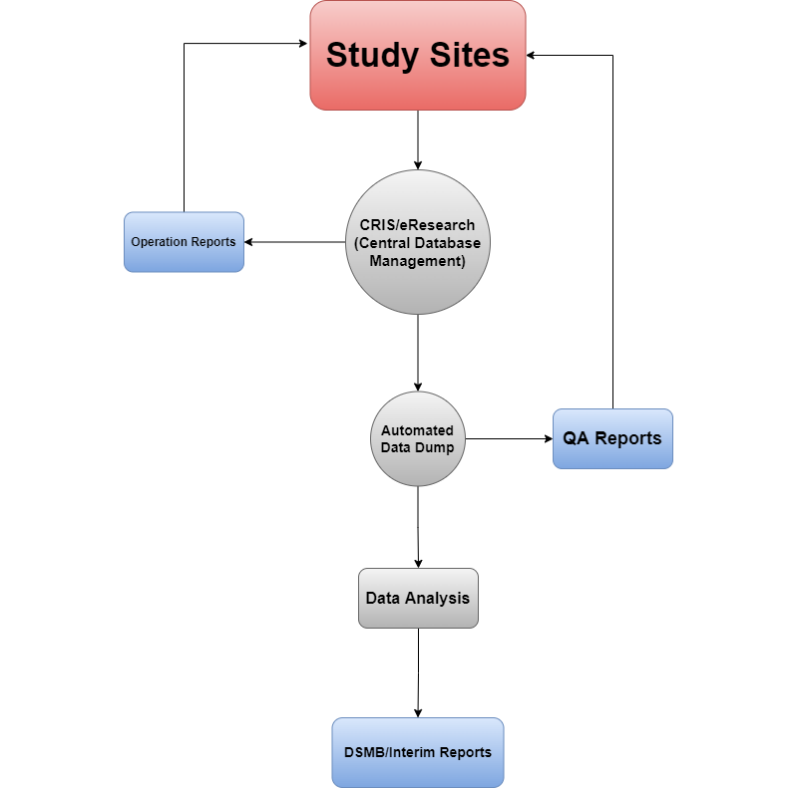
Architecture diagram.

Data harmonization is a significant hurdle for multisite studies but was aided here by having a central database and standardized data entry forms, Data harmonization occurred centrally at the KUMC utilizing CRIS and through the KUMC Biostatistics & Data Science team. Following data entry and validation at individual sites, the study results for each site were validified by regular performance of edit, logic, and range checks by the trial analyst. Queries were then sent to the clinical team at each site in the form of weekly automatically generated emails to resolve any discrepancies. All queries were resolved before the trial analyst created data sets for the interim and final analyses. The finalized study binder was produced in collaboration with the KUMC Director of Research Information Technology and contained copies of the annotated project case report forms, final data dictionary, and copies of electronic data files. This process can be visualized in Figure 1.

All the raw participant information is captured by the site coordinators in real time with the participant in the room. Weekly reports are then generated and automatically pushed to the study team members to help keep them abreast of the study progression. Additionally, automated quality assurance reports help the quality analysis team to verify and address data inconsistences. Finally, the automated data dump is also utilized by the statistical team to perform data analysis, which is then utilized for Data Safety and Monitoring Board (DSMB).

### Study Management

The primary aim of CRIS in study management was to increase procedural uniformity across multiple stages of participant engagement. Research teams at all sites reviewed eligibility criteria, including age, gestation length, and multiple gestation, in CRIS prior to enrollment. After consent, enrollment and randomization was captured using CRIS. Each site was expected to enroll 2-3 participants weekly, and integration and management of accrual data occurred centrally at the KUMC. Each of the 3 sites were given a separate randomization code, and participants at each site were randomized using a Bayesian Adaptive Design detailed by Brown et al [15]. Subsequent adaptations and adapted randomization schedules were appended to each site within CRIS. The ability of CRIS to ensure accrual equality across study sites was assessed using a Gini coefficient. Haidich et al [16] proposed that a Gini coefficient for accrual distribution in multisite trials could provide a standardized approach to assessing accrual disparities. They identified a Gini coefficient of less than 0.2 as suggesting low accrual inequality and calculated a mean Gini coefficient of 0.33 among multisite trials.

### Ethics Approval

The KUMC granted approval under a central institutional review board with reliance by the other institutions (STUDY00003455). The trial was registered on ClinicalTrials.gov (NCT02626299) on December 8, 2015.

## Results

### Results Overview

Features such as electronic data capture, data monitoring, and data validation are common in clinical trial management systems. An additional benefit of implementing the CRIS in this trial came from automated reports for trial accrual, study protocol adherence, and data validation tailored to the challenges of multisite trials. Accrual reports were developed to ensure consistent accrual, accrual equality between sites, and to provide accrual predictions. Study protocol reports were developed to ensure protocol adherence and prepare study teams for upcoming responsibilities. Data validation reports were generated to maintain data integrity and assist in data harmonization. These reports allowed the CRIS to provide additional support to the trial team.

### Trial Accrual Reports

Accrual equality *across* study sites is important in multisite trials as it helps capture a much broader picture, allowing study teams to generalize easily. The calculated Gini coefficient for the ADORE trial was 0.14, indicating a low accrual inequality among sites with 359 patients enrolled from the OSU sites, 489 patients from the KUMC sites, and 252 patients from the UC site. The presence of low accrual inequality in this study suggests that the generalizability of study results was maintained. Enrollment at KUMC began 3 months prior to OSU and 5 months before UC.

**Figure 2:**
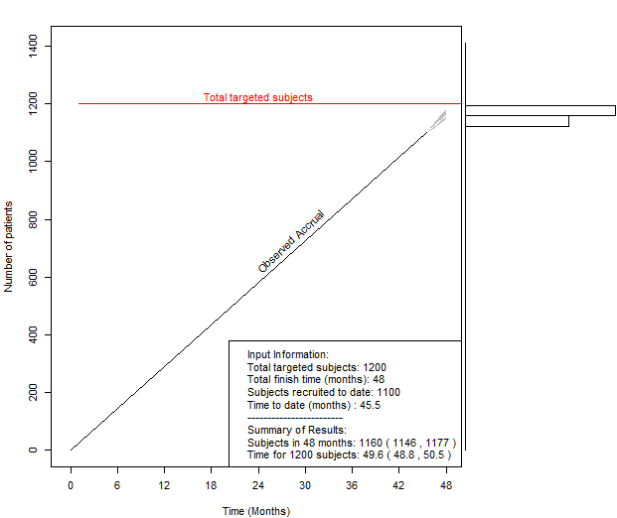
Accrual prediction plot; accrual prediction with visualization plots to demonstrate the predicted completion date with a 95% prediction interval and the posterior predictive distribution. Redline represents the study deadline. The black line represents the current accrual rate.

Successful accrual in the ADORE trial relied heavily on the weekly accrual and delivery reports generated by the CRIS. The reports began with a summary of current enrollment and delivery figures, including predictions for accrual goal achievement and 95% CIs. Participant status was outlined, including participants receiving treatment as well as those who had completed the study or discontinued treatment early. Accrual by site was available to view in the current month (Table 1), past 30 days, and over the course of the entire study. Study primary outcomes such as births in the last 30 days by site (Table 1) and total deliveries by site were also included. Lastly, several plots were available to visualize these figures such as participant accrual and total deliveries (outcomes), overall and by site, and the accrual prediction plot (Figure 2). Accrual reports were generated using R (version 3.6.2; The R Foundation) and included a description of utilized packages. Review of this weekly report became a primary feature of discussion among the team. It kept the team focused and often pointed to early opportunities to adjust recruitment and follow-up tactics to stay on track.

**Table 1:** Accrual by site in the current month, the past 30 days, and overall.

Site		Participants, n
**Current month**		
	Ohio State University	5
	University of Kansas Medical Center	4
**Last 30 days**		
	Ohio State University	7
	University of Kansas Medical Center	10
**Total accrual**		
	Ohio State University	359
	University of Kansas Medical Center	489
	University of Cincinnati	252

### Study Protocol Adherence Reports

An AEs report was generated twice weekly (and immediately for serious AEs) for principal investigators to determine attribution and relatedness. Prompt review of AEs by the principal investigator kept the trial in regulatory compliance and allowed the investigators to quickly see any safety or data entry issues. A principal investigator sign off report that was generated twice monthly included participants who had completed the study and allowed their records to be reviewed for accuracy in a timely manner.

**Figure 3:**
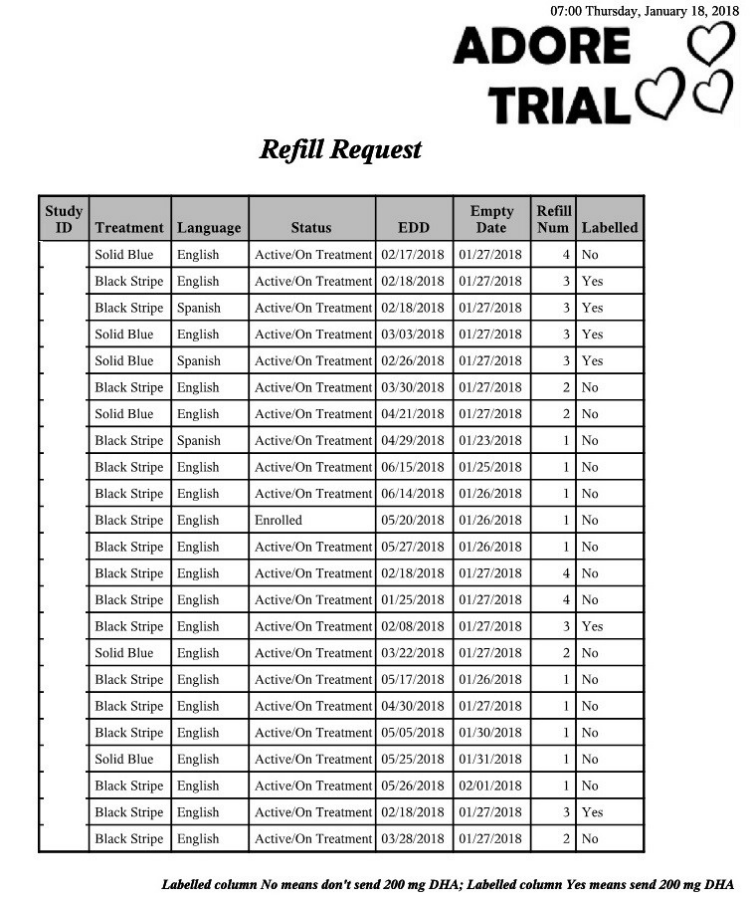
Automated weekly pharmacy refill report. DHA: docosahexaenoic acid

A resupply request report was generated for study coordinators and pharmacists. Resupply request reports detailed upcoming medication refills for study participants (Figure 3). For each study participant, her last medication refill date was recorded along with a date 2 weeks prior to when the current prescription was expected to run out. This allowed time for the prescription to be filled and mailed to the participant and prevented delays in treatment. Study coordinators at all sites benefited from reviewing this report and to anticipate which participants required telephonic or in-person follow-up to ensure refills arrived in good condition.

The delivery watch list report was generated weekly for study teams at each site and made available to all members of the study team. The report included the estimated delivery date for participants who were due to deliver in the next couple of months. The report kept teams aware of participants to watch for in the delivery service so that the collection of necessary delivery blood samples could be ensured.

### Data Validation Reports

Data query reports were generated weekly for each site and identified missing or invalid data on the eCRF so they could be corrected by study teams in the CRIS. As mentioned previously, electronic data capture produces fewer errors and allows missing or invalid data to be quickly and automatically identified [14]. The Data Query reports used in this study leveraged the accessibility of electronically captured data to automate data review processes for significant time-saving during data harmonization.

### Interim Analysis

Eleven interim analyses were conducted during the ADORE trial during which treatment randomization was adjusted in accordance with the Bayesian Adaptive Design. An average of 3-4 days were spent on each interim analysis. Of these days, two of them were working days during which the analyst locked the data, reviewed the data, and updated the randomization table.

### Measures of Benefit

Two primary measures of benefit for the ADORE trial were captured: protocol deviation and loss to follow-up. Protocol deviation is a common occurrence in clinical trials, with an average reporting rate of 0.08 protocol deviations per participant [17]. For 1100 participants, there were 2 protocol deviations in the ADORE trial, constituting a rate of 0.0018 protocol deviations per participant. Of the 1100 participants in the ADORE trial, 68 were lost to follow-up. This constituted 6% of the study participants and was lower than the predicted loss of 15%.

The effect of these systems on study management was to allow a seamless process of data entry, feedback, and communication between study sites. The process of data capture, validation, flagging for inconsistencies or errors, and data correction occurred automatically within a single system. The statistical analysis team at the central site could track each of these processes as they occurred and communicate with study teams completely within CRIS. This eliminated the need to interact with multiple systems for these processes and allowed the CRIS to be used as an encompassing clinical trial data tool.

## Discussion

### Principal Findings

Centralized data capture can be more cost effective, accurate, and efficient than decentralized systems when implemented effectively [18,19]. However, the variability in how centralized data capture is performed presents an operational hurdle for effective implementation. If mismanaged, centralized data capture can exacerbate the complexity that arises from differences between study sites [20]. CTMSs can make centralized data capture more feasible through the availability of key features for multisite management. Unfortunately, many of these systems are either quite expensive or lack the functionality to manage large trials effectively [21].

The primary aim of developing the KUMC CRIS was to design an adaptable CTMS that streamlined the centralized data management process through automated monitoring, verification, integration, and reporting. The ADORE trial presented a unique opportunity through which to test the benefits and limitations of this system for future use. Throughout the ADORE trial, the CRIS was able to ensure the quality of the study data through frequent data quality checks, regularly generated operation reports with little oversight, and ensuring familiarity with CRIS was consistent across systems. One crucial aspect of CRIS implementation in this trial was ensuring that a member from each site travelled to KUMC for training specifically in using this system. This ensured that at least one member of each study team had direct contact with the central data management team, and the CRIS before their site’s study team was trained.

Other web-based CTMSs have been designed for multisite trials, and these systems served as a useful reference to build from when deciding what functionality should be included in the CRIS [22,23]. Durkalski et al [22] detailed a clinical trial management system used in a multisite trial that included functions such as centralized participant enrollment and randomization, real-time reporting of CRF completion rates, and real-time data validation upon data entry. The CRIS system was able to integrate these features as well as accrual tracking and prediction, patient assignment and randomization using the Bayesian Adaptive Design, and centralized training tracking for study staff. One of the chief benefits of sharing the design and structure of these novel systems is that it allows other research teams to begin with a reference for essential functionality and build on that design even further. Development of the CRIS through utilization in other clinical trials will allow us to discover what additional functionality is needed. Further, this study could help other research centers adapt and improve our CRIS design. Thus, large research centers that frequently run multisite trials can help each other become more efficient.

It is important to emphasize that the tools developed for the CRIS were designed to facilitate strong teamwork and communication between teams at all sites. With limited resources at each site, a centralized data management design can allow multisite trials to allocate more resources toward effective trial execution. We would recommend the implementation of similar tools for any research center that frequently acts as a primary site in multisite trials.

Future developments of the CRIS will occur through implementation in subsequent KUMC multisite trials. Aspects of the CRIS such as operational reports and trial accrual reports will be configured for each of these trials in accordance with their unique circumstances. These configured designs can then be used as templates for future trials with similar features, eventually resulting in a library of potential reports that can easily be tailored to each new trial.

As the costs of clinical trials continue to rise, new techniques will be required to manage resources effectively [24]. Multisite trials utilizing centralized data capture are cost-effective because they remove the requirement of data management teams at each site [25]. These designs can, however, present significant challenges to data management and communication between study teams. Several tools can be used to address these challenges, such as automated operational reports, accrual reporting, consistent training, and centralized data validation. Thus, the development of a CTMS specifically for this purpose can provide significant cost savings and efficient trial execution for large research centers. Final data analysis and unmasking randomization took couple of days instead of weeks or months, which is a testament of all the hard work that has been vested by the team from day 1.

### Limitations

While the CRIS system was able to be effectively utilized in the ADORE trial, there was a significant investment required for both development and onboarding.

First, the time required to build the database within CRIS with minimal customization is 3-4 weeks for phase 1, 2, or 3 studies. The database design and data validation systems could also require more time for development if the study protocol is not finalized. This development time may be alleviated as the system is used for more studies and we are able to develop templates for common study design features. At the present, however, this represents a significant time investment. Furthermore, the customization of eCRFs was limited only to the central data management team. The second limitation of this system was the process of standardized training and onboarding. This required a time and labor investment on the part of study teams, and a willingness to learn how to use the CRIS systems. As CRIS is updated with subsequent trials, the training material will also need to be updated. Because study personnel at each site need to be trained on the same material for standardization purposes, this could require additional time and resources.

### Conclusions

This study shows that multicenter trial success is dependent on prompt orchestration. Utilizing a platform to ease the execution steps is crucial, which the team at KUMC has demonstrated through the use of the CRIS.

## Appendix

Multimedia Appendix 1 List of Case Report Forms used for ADORE study.

## Abbreviations

AE: adverse event
CRIS: Comprehensive Research Information System
CTMS: clinical trial management system
DHA: docosahexaenoic acid
DSMB: Data Safety and Monitoring Board
eCRF: electronic case report form
ICH GCP: International Council for Harmonization Guideline for Good Clinical Practice
KUMC: University of Kansas Medical Center
RCT: randomized controlled trial
WCG: Western Institutional Review Board–Copernicus Group, Inc
