# Supplementary figures and images for: The Successful Synchronized Orchestration of an Investigator-Initiated Multicenter Trial Using a Clinical Trial Management System and Team Approach: Design and Utility Study
Source: JMIR Form Res. 2021 Dec 22;5(12):e30368. doi: 10.2196/30368 (PMC8734918; doi:10.2196/30368)

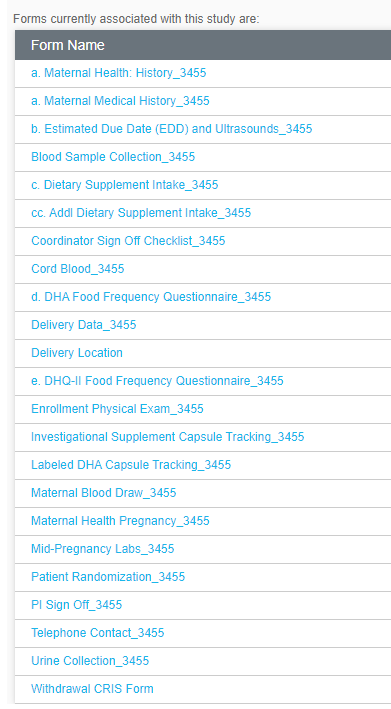

Supplement: Multimedia Appendix 1 [file formative_v5i12e30368_app1.docx]
